# Homozygous Mutation in the QARS1 Gene Causing Developmental Epileptic Encephalopathy in Siblings in the Southeast Asian Region: An Interesting Case Report and Discussion

**DOI:** 10.7759/cureus.78333

**Published:** 2025-02-01

**Authors:** Preeti Srivastava, Md W Uddin, Kumar Diwakar, Summet Biswal, Aishwarya Senapati

**Affiliations:** 1 Department of Paediatrics, Tata Main Hospital, Jamshedpur, IND; 2 Department of Paediatrics, Manipal-Tata Medical College, Jamshedpur, IND; 3 Department of Paediatrics, Tata Main Hospital, Jamshedhpur, IND; 4 Department of Paediatrics, Manipal-Tata Medical College, Jamshedhpur, IND; 5 Department of Paediatrics, Manipal Tata Medical College, Jamshedpur, IND

**Keywords:** developmental and epileptic encephalopathy, drug-refractory seizures, global developmental delay (gdd), microcephaly, qars gene mutation

## Abstract

Developmental epileptic encephalopathy (DEE) refers to conditions where cognitive functions are impacted both by seizures as well as interictal epileptiform activities and the neurobiological processes involved. They lead to early onset refractory epilepsy causing progressive decline in cerebral function, developmental delay, and significant EEG changes. Glutaminyl-tRNA synthetase (QARS) is encoded by the *QARS* gene and its mutation has been implicated as one of the causes of DEE. We report two cases of siblings with *QARS* mutation-associated DEE, severe global developmental delay, and microcephaly. The babies were born of a non-consanguineous marriage. All basic investigations and metabolic tests of both siblings were normal. Magnetic resonance imaging of the brain of both siblings showed loss of cerebral white matter. Electroencephalography showed multifocal epileptiform discharges in the left temporo-occipital and right frontal regions. Both siblings suffered from refractory epilepsy. Genetic tests and clinical exome sequencing revealed homozygous missense variation in exon 2 of the *QARS 1* gene in both the siblings, and heterozygous states for their parents. There is a wide range of aetiologies for DEE with microcephaly, which have overlapping clinical presentations. With growing awareness and availability of genetic tests, it has become possible to do workups for complex neurological disorders. Establishing precise etiology helps in outlining the treatment (if available) and providing a prognosis to parents. It also plays a critical role in planning future pregnancies.

## Introduction

Developmental epileptic encephalopathy (DEE) refers to conditions where cognitive functions are impacted by seizures and interictal epileptiform discharges. They lead to early onset refractory epilepsy causing progressive decline in cerebral function, developmental delay, and significant EEG changes [1]. New genetic variants associated with DEE are increasingly being recognized. Glutaminyl-tRNA synthetase1 (QARS1) encoded by *the QARS1* gene catalyzes the attachment of amino-acid to their cognate tRNA and helps in maintaining translational fidelity which influences the termination of branching at the dendritic level [2]. Variants in *the QARS1* gene are implicated as one of the causes of DEE [3].

In 2014, Zhang et al. were the first to identify *a QARS1 *gene mutation-associated intractable epilepsy of neonatal onset, severe intellectual disability, microcephaly, and cerebral and cerebellar atrophy in four individuals from two unrelated families [4]. So far, the literature shows 25 patients with *QARS1-*related disease with the occurrence being rarer in siblings. Out of 25 cases, 19 were associated with compound heterozygous mutation while six were associated with homozygous mutation of *the QARS1 *gene [3,5]. Here, we report a case of two siblings from India having DEE, progressive microcephaly, and intractable epilepsy due to homozygous *QARS1* mutation.

## Case presentation

**Figure 1:**
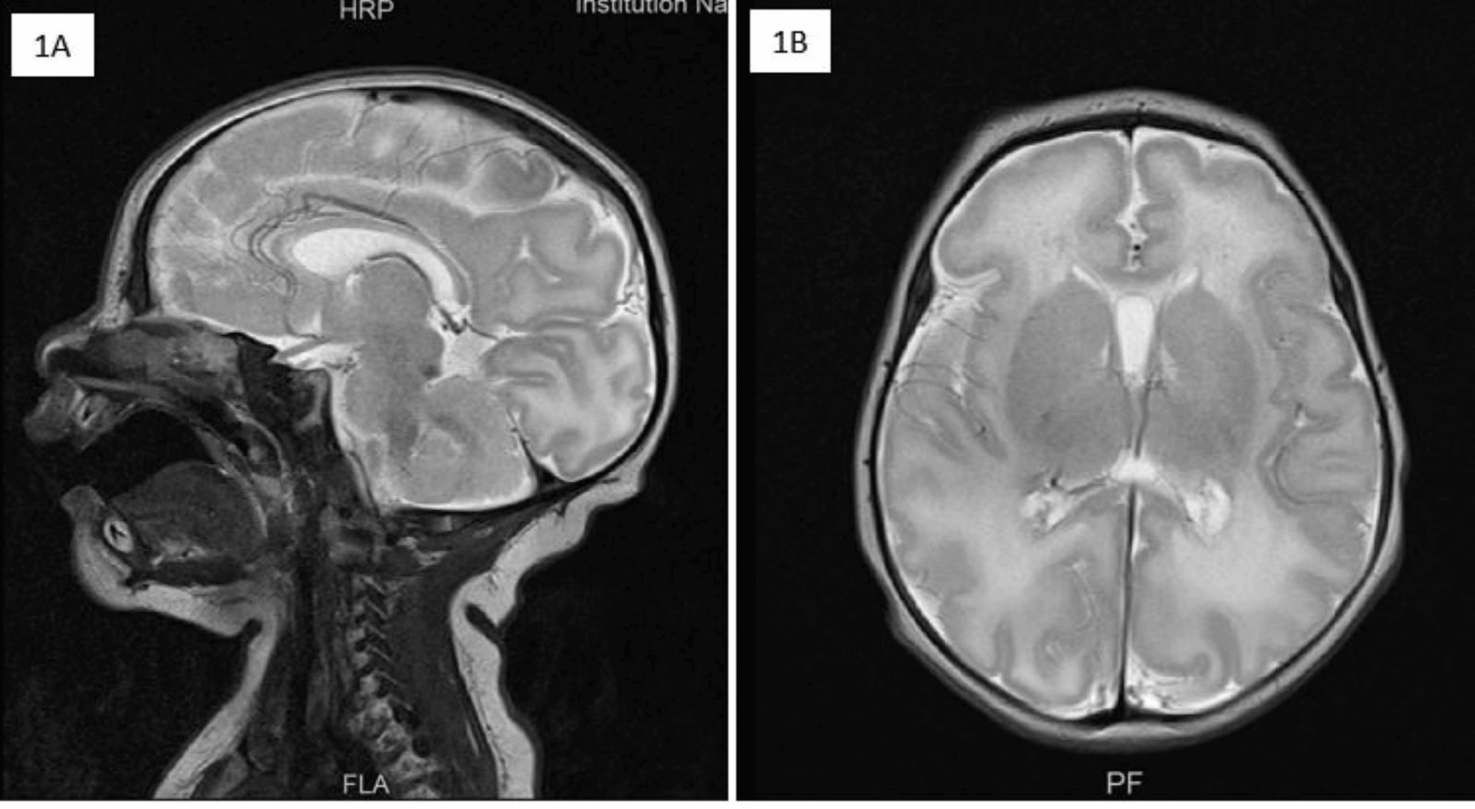
(A) Mid sagittal T2 weighted scan shows presence of normal brain parenchyma without any evidence of atrophy; (B) Axial T2-weighted scan at the level of lateral ventricles and deep grey nuclei shows normal bilateral supratentorial brain parenchyma

The index patient, a six-day-old female baby of non-consanguineous parents, presented with multiple seizures. Seizure semiology comprised deviation of eyes and head towards the right side with clonic movements of all limbs. There was no history of fever, vomiting, refusal to feed, or adverse perinatal events. This baby was born by normal vaginal delivery, cried immediately after birth, and started feeding within half an hour of birth. On admission, the baby’s general and systemic examinations were normal. Neurologically, her primitive reflexes were intact with normal tone and deep tendon reflexes. Anthropometry revealed a weight of 3750 gm (85th percentile), length of 49 cm (50th percentile), and head circumference of 32 cm (-2 z score as per WHO chart). Injection levetiracetam and empirical antibiotics were started. A full septic workup including CSF examination, serum electrolytes, calcium, magnesium, and blood sugar were normal. Next, screening for inborn errors of metabolism didn’t show any abnormality. MRI brain, done on the seventh day of life, was also normal (Figure 1). Routine EEG done at first admission was normal. As the seizures stopped, the baby was discharged on oral levetiracetam (20 mg/kg/day).

Thereafter, the baby was admitted thrice in the next five months with breakthrough seizures and anti-seizure medicines (ASMs) were titrated with the addition of more ASMs like sodium valproate, oxcarbazepine, topiramate, and clobazam, in that order. Intermittently, the child also received phenytoin for acute seizures. Seizure semiology remained the same in the form of tonic deviation of eyes with focal onset to secondary tonic-clonic seizures. Usually, seizures were brief, lasting one to two minutes, but, other times, would be prolonged, lasting 5-10 minutes requiring intravenous lorazepam intranasal midazolam to abort it.

**Figure 2:**
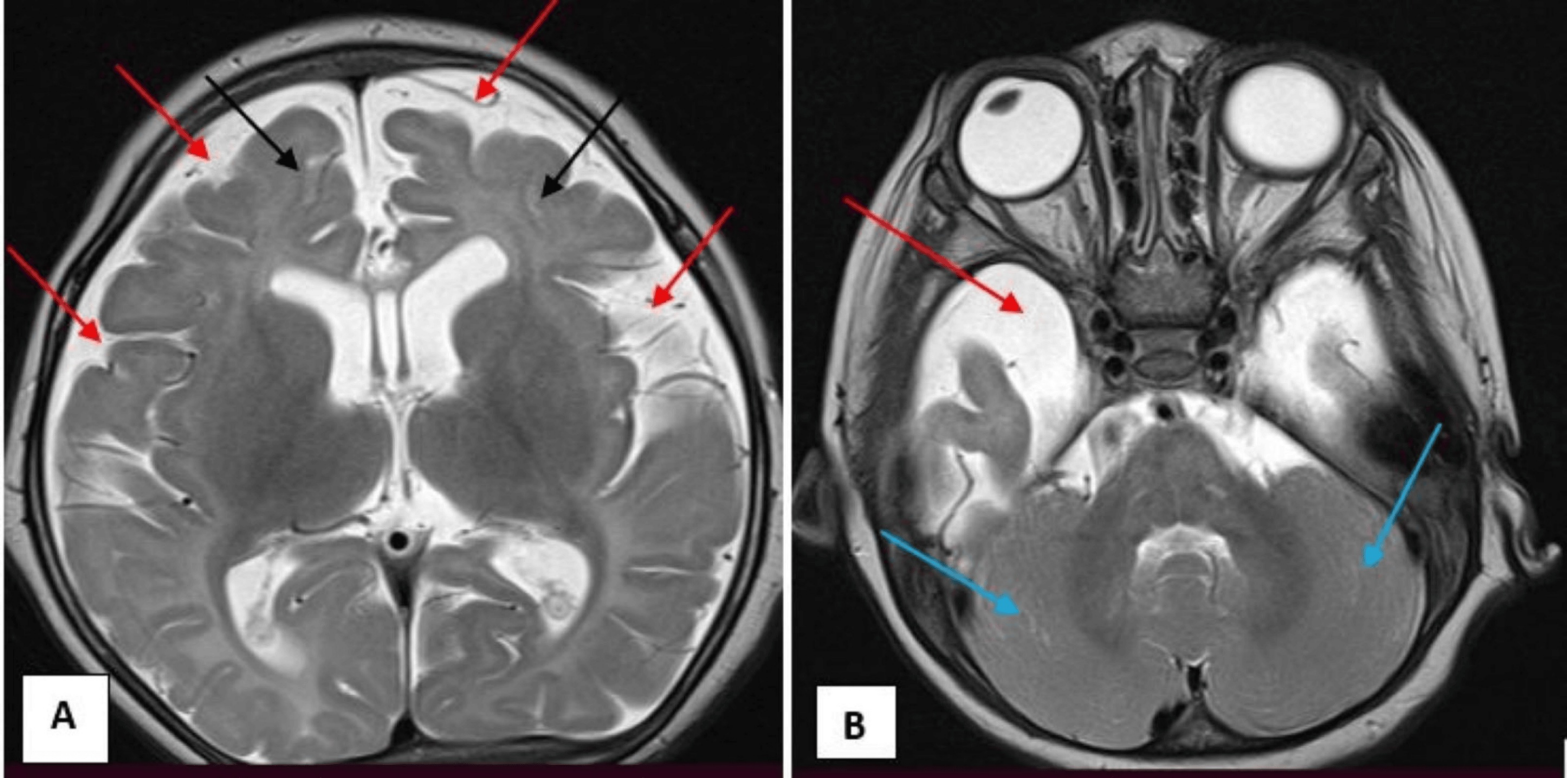
Axial T2W sequence shows (A) diffuse cerebral atrophy (black arrow) at the level of lateral ventricles and increased CSF (cerebrospinal fluid) spaces (red arrow), and (B) preserved volume of the bilateral cerebellar hemispheres (blue arrow) at the level of posterior cranial fossa

As mentioned, in the first five months of life, the baby had frequent breakthrough seizures and was put on five ASMs but from the sixth month onwards, the seizures appeared to stabilize with significantly reduced frequency (once a week or fortnight), duration (lasting few seconds), and intensity. Serial EEGs, done at the second, sixth, and eighth month of age showed abnormalities in the form of epileptiform discharges in the central region (Cz) at the second month and multifocal epileptiform discharges in left temporo-occipital (T3, T5, O1) and right frontal regions (F8) at the sixth and eighth months of life. Magnetic resonance imaging (MRI) of the brain done at the age of 12 months showed diffuse cerebral atrophy with preserved volume of the bilateral cerebellar hemispheres (Figure 2).

There was significant family history as the index patient’s elder sister had neonatal seizures starting at 40 days of life. She was worked up extensively for the cause of seizures. MRI done at three months of age showed cortical atrophy, thinning of the corpus callosum, and supra tentorial white matter volume loss. EEG showed epileptiform discharges in bilateral occipital regions. The baby also had global developmental delay, microcephaly, pyramidal signs, and worsening epilepsy (was on five ASMs) and died at the age of two years and three months. The genetic test of the sibling, clinical exome sequencing, revealed homozygous missense variation in exon 2 of the *QARS1* gene (chr3:g.49141823; G/A; Depth: 172x) that results in the amino acid substitution of tryptophan for arginine at codon 67 (p.Arg67Trp; ENST00000306125) and inherited in an autosomal recessive manner. Based on the clinical presentation of the index patient and the sibling’s background, a clinical exome sequencing of the patient along with both parents was done. The test revealed homozygous mutation in the* QARS1* gene (chr3: c.199C>T; G/A; Depth: 172x) that results in the amino acid substitution of tryptophan for arginine at codon 67 (p.Arg67Trp; ENST00000306125.12) for the baby and heterozygous mutation in *QARS1* gene for the parents (Table 1).

**Table 1:** Clinical exome sequencing of index case, sibling, and parents OMIM: Online Mendelian Inheritance in Man

	Index case	Elder sibling	Mother	Father
Gene (Transcript)	*QARS1* (-) (ENST00000306125.12)	*QARS1* (-) (ENST00000306125.12)	*QARS1* (-) (ENST00000306125.12)	*QARS1* (-) (ENST00000306125.12)
Location	Exon 2	Exon 2	Exon 2	Exon 2
Variant	c.199C>T (p.Arg67Trp)	c.199C>T (p.Arg67Trp)	c.199C>T (p.Arg67Trp)	c.199C>T (p.Arg67Trp)
Zygosity	Homozygous	Homozygous	Heterozygous	Heterozygous
Disease (OMIM)	Progressive Microcephaly with seizures and cerebral and cerebellar atrophy	Progressive Microcephaly with seizures and cerebral and cerebellar atrophy		
Inheritance	Autosomal recessive	Autosomal recessive	Autosomal recessive	Autosomal recessive
Classification	Uncertain Significance	Uncertain Significance	Uncertain Significance	Uncertain Significance

Reference to OMIM (Online Mendelian Inheritance in Man) suggested that microcephaly, progressive, seizures, and cerebral and cerebellar atrophy (OMIM#615760) are caused by homozygous or compound heterozygous mutations in the *QARS1 *gene (OMIM*603727). This disorder is characterized by progressive microcephaly with seizures, cerebral and cerebellar atrophy, and severe neurodevelopmental and neurodegenerative disorder with onset in the first days or months of life. Patients are born with microcephaly and soon develop intractable seizures, resulting in profoundly delayed development and hypotonia. Although this variant was reported as "variant of unknown significance (VUS)", a clear genotype and phenotype match was seen in the index patient as well as in the deceased sibling, and hence the variant was considered clinically significant. Both parents carried a heterozygous variant of *the QARS1 *gene and were asymptomatic and healthy. Thus, based on the genetic test result, a complete diagnosis of *QARS1*-related DEE and microcephaly was established in the index patient.

## Discussion

The enzyme, QARS, encoded by the *QARS* gene, is a member of the aminoacyl-tRNA synthetases (aaRSs) family. aaRSs attach amino acids precisely to correct tRNA to maintain translational fidelity [6]. There are 37 aaRSs encoded by the human genome of which 18 aaRSs operate exclusively in the cytoplasm while 17 are limited to mitochondria and two are common in both [7,8].

The first association between aaRSs and diseases like Charcot-Marie-Tooth disease type D and distal spinal muscular atrophy type V was identified by Antonellis et al. way back in 2003 and since then, various disease-causing mutations in the *aaRSs* gene have been identified [9]. Mutation in mitochondrial and cytoplasmic aaRSs-encoding genes covers a wide spectrum of illnesses like mitochondrial myopathy, sensorineural deafness, Charcot-Marie-Tooth neuropathies (GARS), etc. [10-12]. Zhang et al., in 2014, first described *QARS* gene mutation in four individuals from two unrelated families with progressive microcephaly, intractable seizures, and cerebral-cerebellar atrophy. They also demonstrated that QARS-/- fish have smaller eyes and brains due to significantly more apoptosis in these organs [4]. The genotype-phenotype association in QARS encephalopathy was described in a cohort of 22 patients by Johannesen et al. [2]. Microcephaly was an important clinical feature present in all patients at six months of age. Developmental delay was characteristic in relation to sitting, walking, or talking and it was moderate to severe. They noted pharmaco-resistant epilepsy with onset as early as the neonatal period. Other features were the presence of irritability, hypertonia, spasticity, and stereotypic movements. On MRI scans of the brain, there was early-onset progressive atrophy of the cerebral cortex and cerebellum [2]. Another study by Kodera et al., in 2014, described two affected siblings with compound heterozygous *QARS *mutation, with key features of signs of excitement like crying, bending backward, excessive sweating, hypertonicity, and sleep disturbance [13].

Reviewing the literature available online and from previously published case reports of *QARS *gene mutation, the current case report is the first of its kind showing *QARS1* gene mutation in siblings from the Southeast Asian region. Our case also presented in the early neonatal period with refractory seizures and developed progressive microcephaly with global developmental delay. The first MRI brain, done in the early neonatal period was normal. A second MRI brain was done at 12 months of age which showed diffuse cerebral atrophy. Genetic tests and clinical exome sequencing of the baby and her parents were done in view of the history of *QARS1* mutation in the elder sibling. Homozygous mutation in the child and heterozygous mutation in the parents was found using next-generation sequencing technology. In the literature, it has been observed that homozygous mutation in the *QARS1* gene is far rarer than compound heterozygous mutation.

The prognosis, in cases of *QARS* with homozygous mutation, appears slightly better than that in compound heterozygous mutation [2]. But in our case, the elder sibling of the index case succumbed at the age of two years and three months. A multidisciplinary team approach is the cornerstone of the management. Special attention should be given to nutritional deficiencies due to feeding difficulties and aspiration pneumonia due to microaspirations due to recurrent seizures along with speech therapy. There should be emotional support for the parents to cope with the difficult time. Genetic counseling of the parents is essential to prevent recurrence in subsequent pregnancies as, in this case, they were heterozygous carriers of the mutated gene.

## Conclusions

There is a wide range of etiologies for developmental epileptic encephalopathy with microcephaly, which have common and overlapping clinical presentations. There is growing awareness and availability of genetic tests with advances in the field of genetics, which help in a better workup of complex neurological cases. A precise and timely diagnosis is not just very critical for outlining the course and treatment (if available) and prognosis of the disease to parents, but also plays a critical role in planning future pregnancies.
